# Microscopic images dataset for automation of RBCs counting

**DOI:** 10.1016/j.dib.2015.08.006

**Published:** 2015-08-20

**Authors:** Sherif Abbas

**Affiliations:** a Ain Shams University, Cairo, Egypt; b Middle East Technical University, Ankara, Turkey

## Abstract

A method for Red Blood Corpuscles (RBCs) counting has been developed using RBCs light microscopic images and Matlab algorithm. The Dataset consists of Red Blood Corpuscles (RBCs) images and there RBCs segmented images. A detailed description using flow chart is given in order to show how to produce RBCs mask. The RBCs mask was used to count the number of RBCs in the blood smear image.

## Specifications table

**Table:** 

Subject area	*Biology*
More specific subject area	*Medical image analysis*
Type of data	*Light microscopic images, graph, figure.*
How data was acquired	*Light Microscope, The images acquired using Panasonic WV-CP220 series digital video camera. With Automatic Tracing White (ATW) balance and built in automatic gain control (AGC). Minimum illumination of 0.5 lx at F0.75 and signal-to-noise ratio of 46 dB by employing a 1/3 in. interline transfer CCD image sensor with 512(H)×582(V) pixels. And the output signal feed to TV tuner in a computer resulting of 24-bit color image of 332(H)×252(V) pixels which is suitable resolution to capture the cell and its nuclear material.*
Data format	*Raw, segmented, masked images.*
Experimental factors	*N/A*
Experimental features	*Blood smears stained with Leishman stain.*
*Images acquired using light microscope connected to digital camera.*
*Images converted to their three colors components.*
*Initial Segmentation based on green component.*
*Final RBCs segmentation and counting based on the histogram of objects areas in the image.*
Data source location	*Images collected in Biophysics lab. At Ain Shams University, Cairo, Egypt.*
Data accessibility	*Data is with this article.*

## Value of the data

**Table:** 

• To describe an easy segmentation method for RBCs images. • To produce a cheap and accurate RBCs counting computer system. • The images dataset is very useful for any further RBCs medical image analysis.

## 1 Experimental design, materials and methods

The digital camera was attached to light transmission microscope with 10× objective magnification and no eye pieces. The blood smear has been illuminated by 50 W halogen lamp and the image focused by normal microscopic focusing system in order to capture high resolution image of the stained blood smear.

**Fig. 1:**
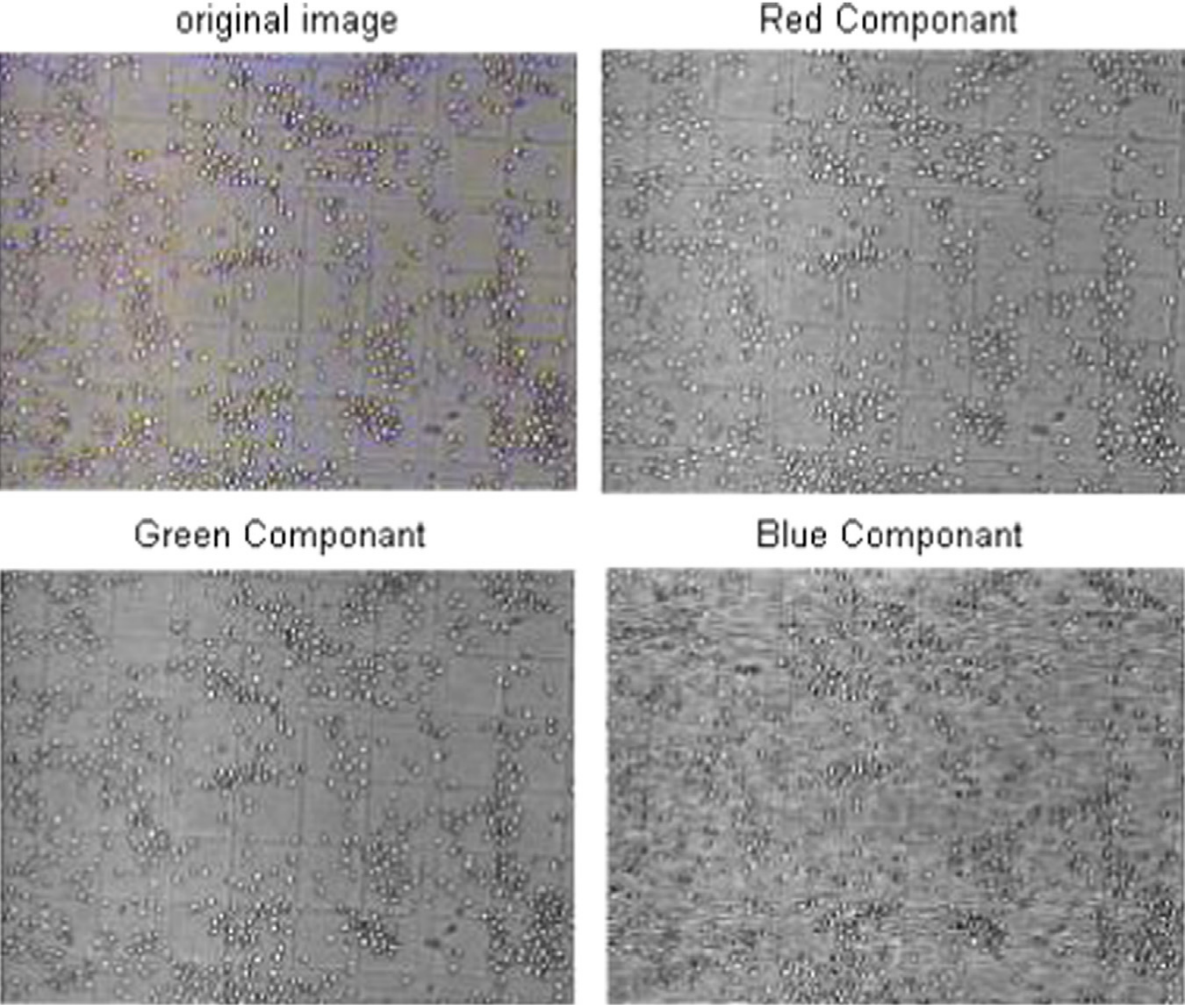
Color components of RBCs.

**Fig. 2:**
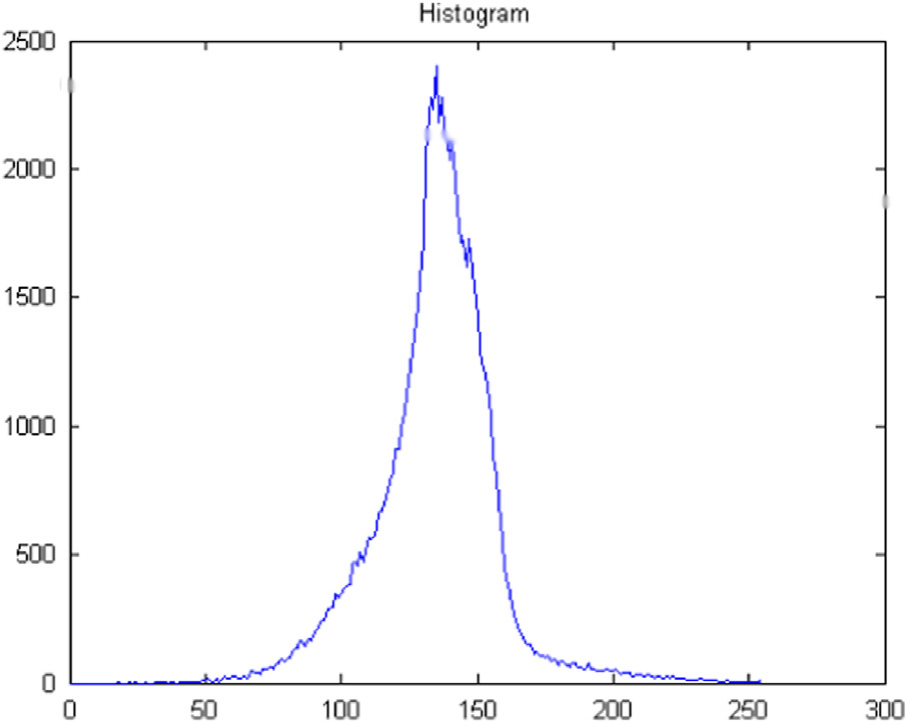
Histogram of green component.

**Fig. 3:**
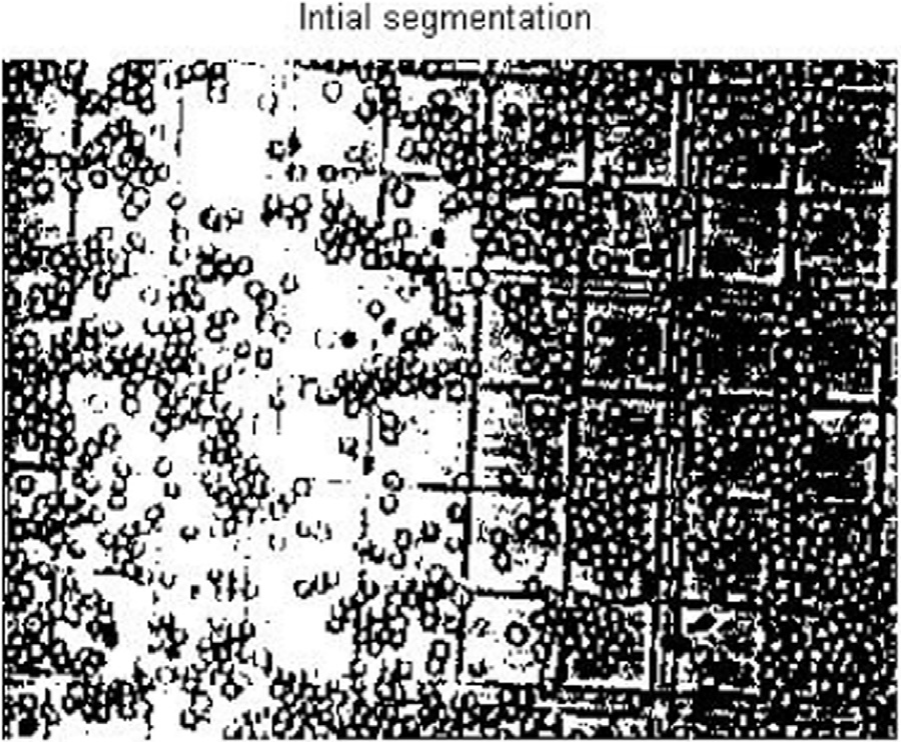
Initial segmentation.

**Fig. 4:**
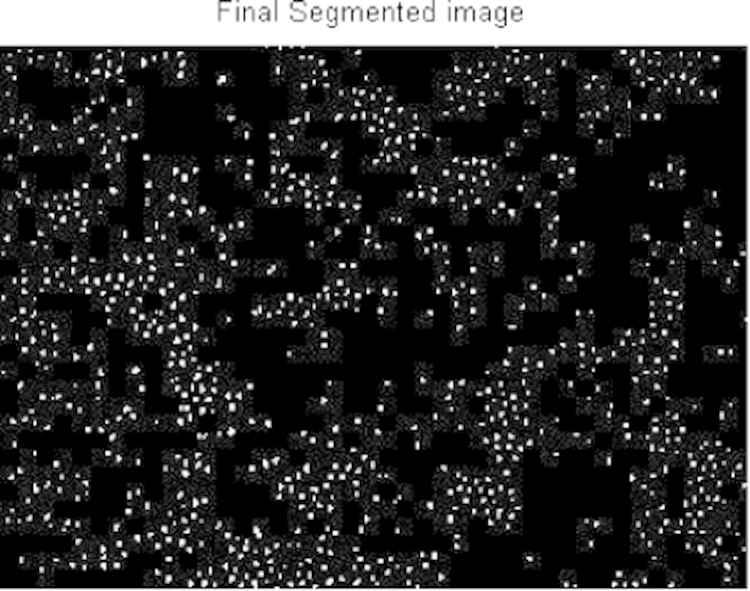
Final segmentation.

**Fig. 5:**
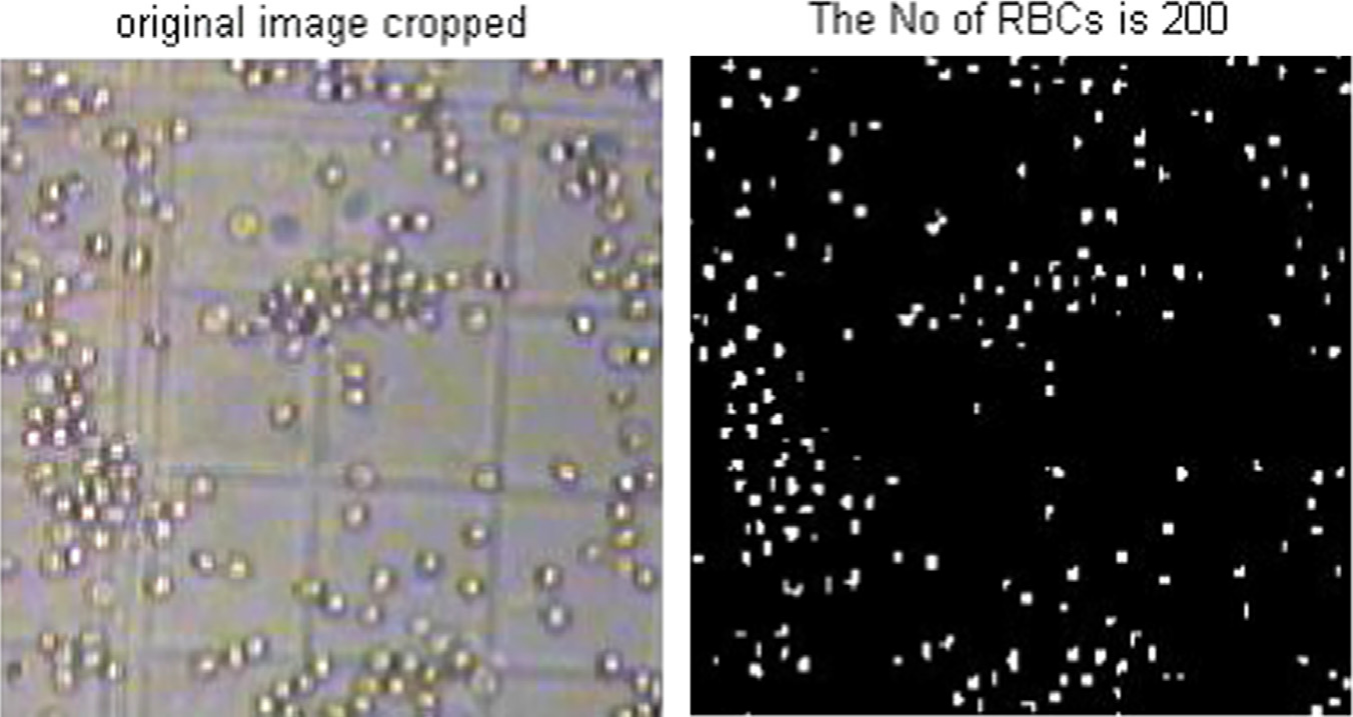
a. Original cropped image and b. Binary cropped image.

**Diagram 1:**
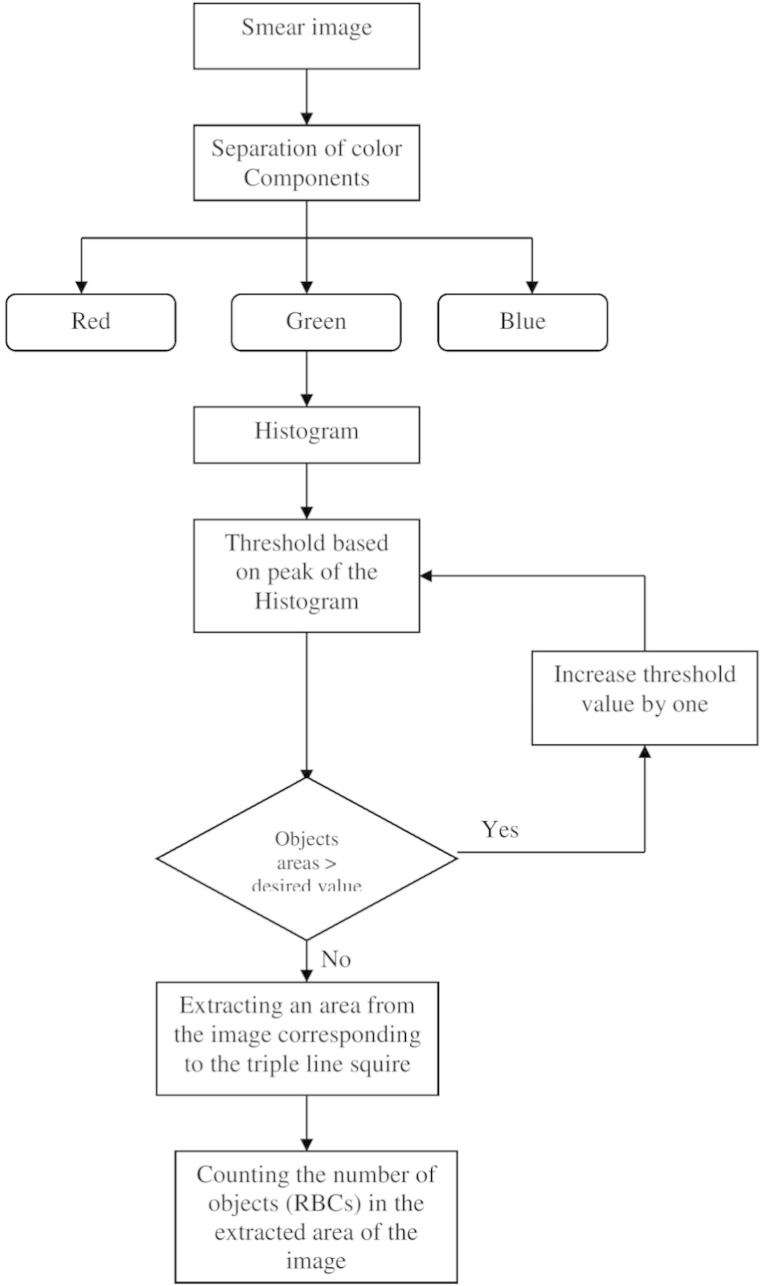
System scheme.

The acquired images subjected to segmentation process as a part of the date reduction stage involves the partitioning of the image plane into meaningful parts [1,2]. In this work a system scheme (Diagram 1) is proposed to segment both the RBCs form the background and the counting area which indicated by the triple line square on the glass slide [3]. The full description of the segmentation scheme is given as follows: At the first step, the captured smear image split into its three color component bands (red, green and blue) as shown in Fig. 1. The three grayscale components are examined and we note that the green components show a clear image of RBCs [4]. At the second step, the histogram green component has been performed and the initial threshold value taken as gray level corresponding to the peak of the histogram as shown in Fig. 2. At the third step, the areas of the objects on the threshold image has been determined which shows that the most common areas are less than 10 pixels corresponding to the area of the RBCs. The objects that have areas larger than this value (10) are due to inaccurate threshold as shown in Fig. 3. Based on this, if there is any object which has an area greater than 10 the feedback increases the value of threshold by one until no longer object has an area greater than 10 as shown in Fig. 4. At the fourth step, extracting the counting area by calibrating the cropped image to be the same area of the triple line square at that system setup as shown in Fig. 5. Finally, the number of objects in the extracted area determined which is corresponding to the number of RBCs in that area. A complete set of images, their RBCs counts and segmentations are included as supplementary material to this article.
